# Supplementary material for: Superdislocations and point defects in pyrochlore Yb2Ti2O7 single crystals and implication on magnetic ground states
Source: Sci Rep. 2018 Nov 21;8:17202. doi: 10.1038/s41598-018-35283-w (PMC6249211; doi:10.1038/s41598-018-35283-w)
Supplement: Supplementary file 1 — Supplementary Information [file 41598_2018_35283_MOESM1_ESM.pdf]

Superdislocations and point defects in pyrochlore $Yb_2Ti_2O_7$ single crystals and implication on magnetic ground states

Zahra Shafieizadeh[1,+], Yan Xin[2,+,*], Seyed M. Koohpayeh[3], Qing Huang[4], & Haidong Zhou[4]

[1] Department of Physics, Florida State University, Tallahassee, FL 32311, USA

[2] National High Magnetic Field Laboratory, Florida State University, Tallahassee, FL 32310, USA

[3] Institute for Quantum Matter, Department of Physics and Astronomy, Johns Hopkins University, Baltimore, MD 21218, USA

[4] Department of Physics and Astronomy, University of Tennessee, Knoxville, TN 37996, USA

+These two authors contributed equally to this work.

* Correspondence and requests for materials should be addressed to Y.X. (email: xin@magnet.fsu.edu)

Contact information:

Dr. Yan Xin

National High Magnetic Field Laboratory

Florida State University

1800 E. Paul Dirac Drive, Tallahassee, FL 32310

USA

Phone: (850) 644-1529

e-mail: xin@magnet.fsu.edu

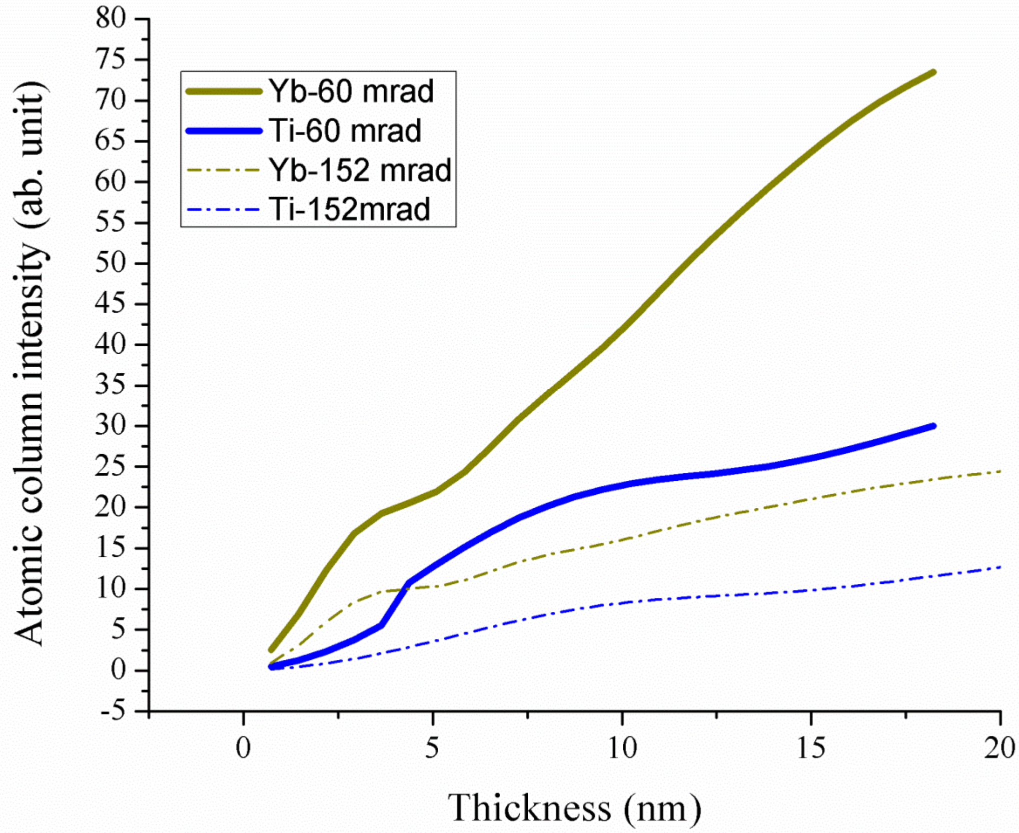

Figure S1 Yb and Ti atomic column intensity vs sample thickness with different inner collection-angle. The solid curve corresponds to the experimental condition used. This calculation confirms that the intensity difference for Yb and Ti columns is mainly due to the atomic number effect rather than diffraction contrast effect.

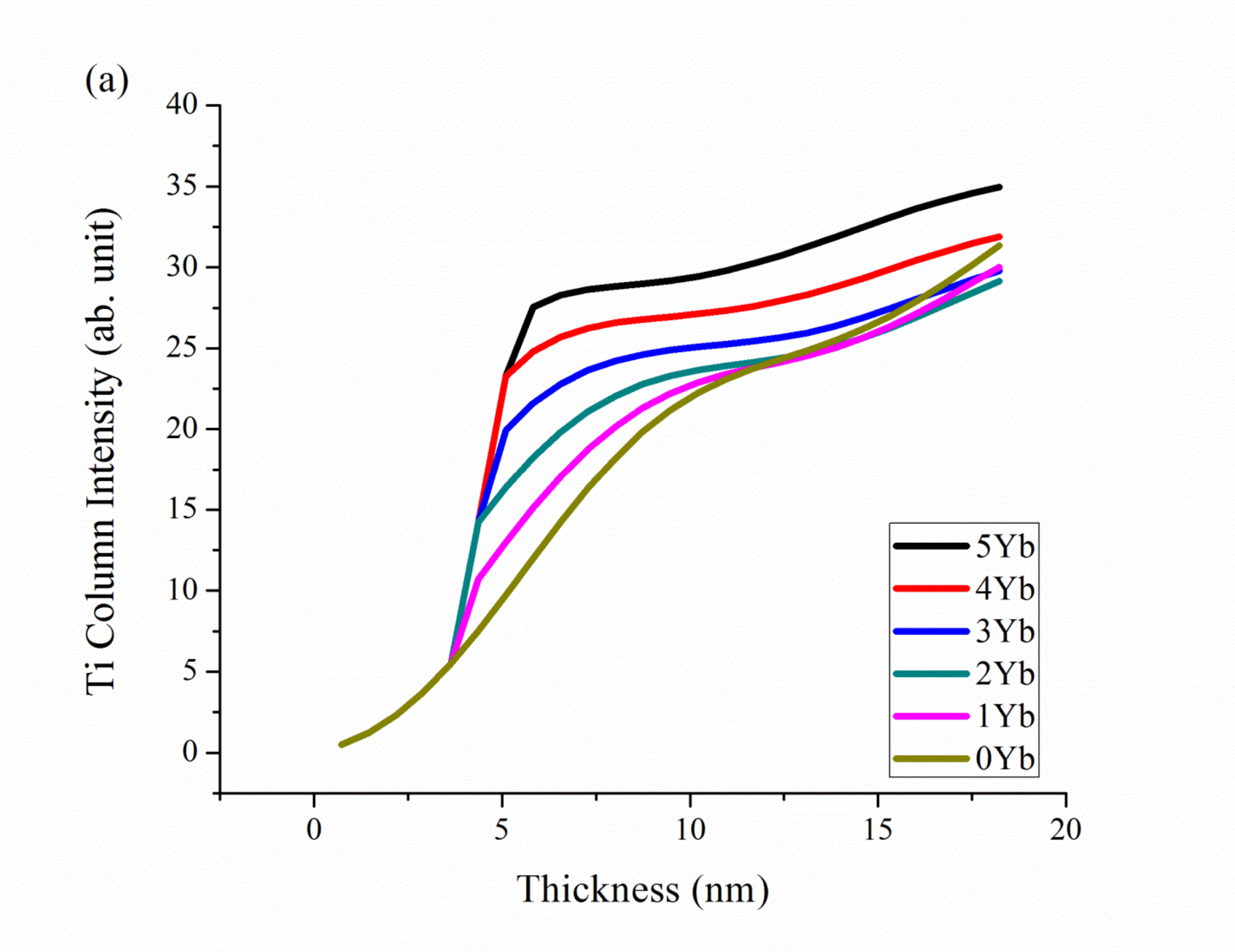

Figure S2 (a) Ti column intensity vs thickness curve for one to five substitution Yb atoms at 3.65 nm below surface;

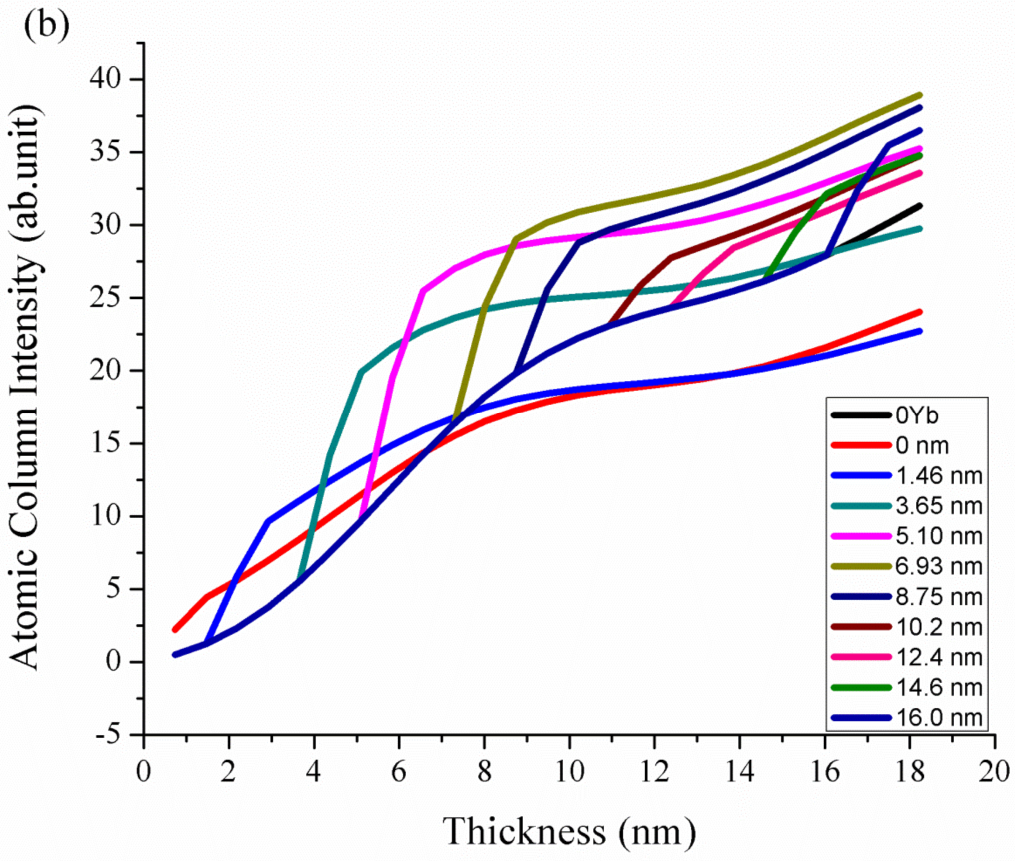

Figure S2 (b) Ti atomic column intensity vs thickness curve for 3 substitution Yb atoms inside Ti column at different depths below sample surface.

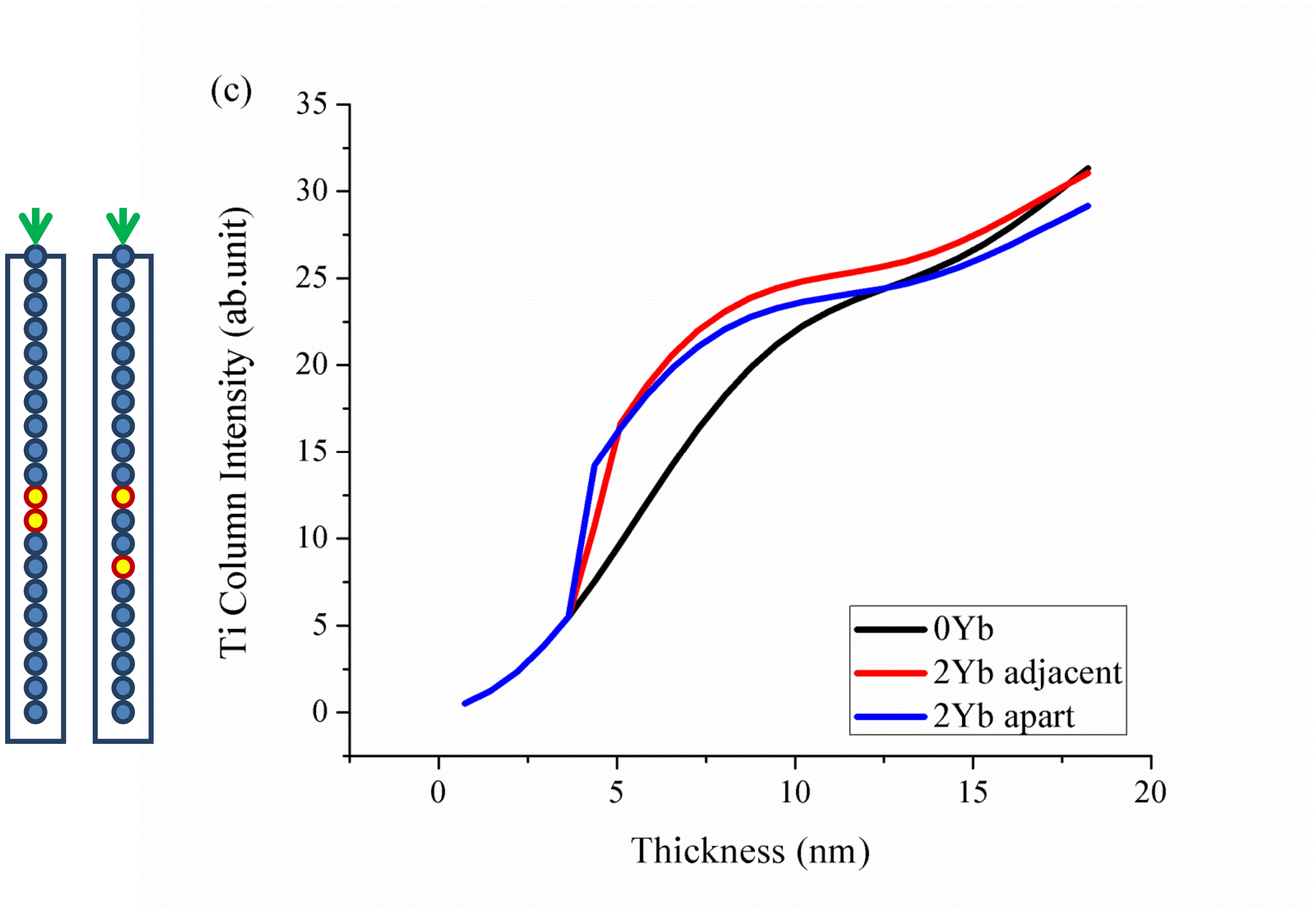

Figure S2 (c) Ti atomic column intensity vs thickness curve for 2 substitution Yb atoms inside Ti column adjacent to each other and apart as illustrated in sketch on the left;

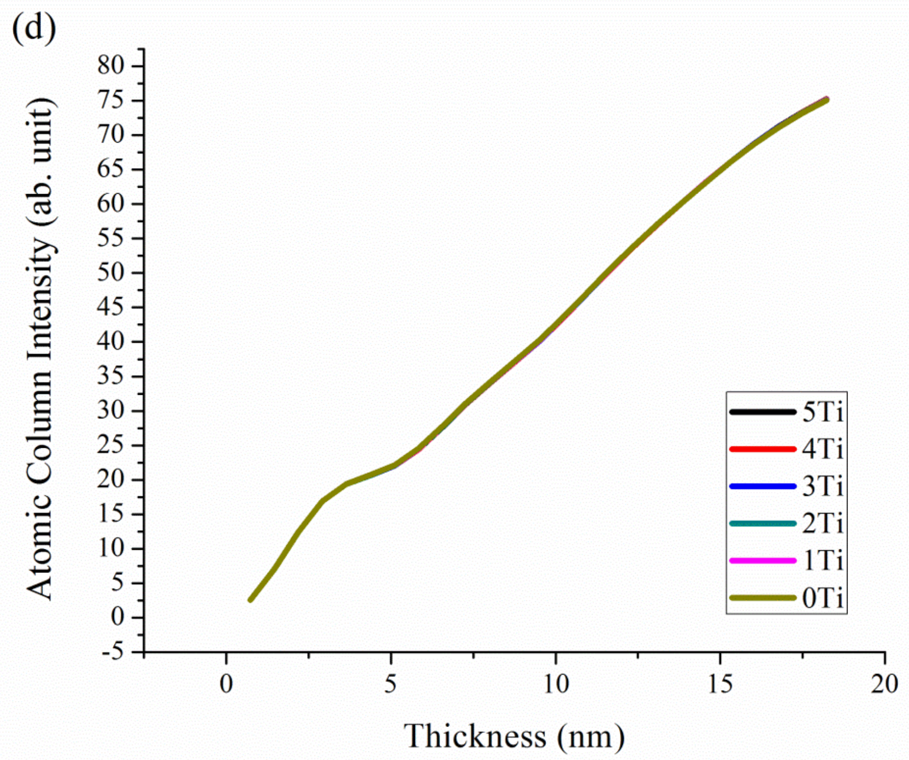

Figure S2 (d) Yb atomic column intensity vs thickness curve of one to five Ti substitution into Yb columns. All five curves overlapping on top of each other.
